# Janus-faced Mitofusin 2 (MFN2): mitochondria-endoplasmic reticulum shaping and tethering functions unveiled

**DOI:** 10.1038/s41392-023-01730-y

**Published:** 2024-01-01

**Authors:** Camilla Aurora Franchino, Elisa Motori, Matteo Bergami

**Affiliations:** 1 Institute for Biochemistry, University of Cologne, 50674 Cologne, Germany; 2 Cologne Excellence Cluster on Cellular Stress Responses in Aging-Associated Diseases (CECAD), Faculty of Medicine and University Hospital Cologne, University of Cologne, 50931 Cologne, Germany; 3 Center for Molecular Medicine, 50931 Cologne, Germany

**Keywords:** Cell biology, Biochemistry

In a recently published study in *Science*, Naòn et al. report two uncharacterized splice variants of Mitofusin 2 (*MFN2*) that specifically shape and tether the endoplasmic reticulum (ER) to mitochondria.^1^ This work sheds new light on the pleiotropic effects previously ascribed to *MFN2* loss-of-function mutations, and has important implications for associated metabolic and neurological diseases.

Mitochondria are dynamic organelles undergoing extensive communication with the ER via so-called mitochondria-ER contact sites (MERCs).^2^ As for other types of contact sites, MERCs play essential roles in transducing cellular stimuli into organelle-specific, compartmentalized signaling and metabolic functions. These functions include Ca^2+^ homeostasis, lipid metabolism, membrane dynamics (e.g., formation of autophagosomes), reactive oxygen species (ROS)-mediated signaling, apoptosis and inflammatory signals.^2^ Assembly and maintenance of MERCs is believed to be mediated by several structural tethers that accumulate at the interface between mitochondria and the ER, thereby anchoring the two organelle membranes to ensure proximity of ~10–30 nm. The crucial role of MERCs in mammalian cells is emphasized by the growing evidence suggesting that MERC dysregulation is a common hallmark of several diseases, including neurodegeneration.

Being anchored to the mitochondrial outer membrane (OMM) by two transmembrane domains, the dynamin-like GTPase MFN2 plays a major role in regulating fusion dynamics. Consistent with this model, loss-of-function Mitofusin 2 mutations in various settings in vitro and in vivo lead to disrupted mitochondrial morphology and, in the long-term, ensuing mitochondrial dysfunction.^3^ However, on account of its presumptive dual localization on the ER, MFN2 has also been proposed to act as structural tether by forming homo- and heterotypic complexes with MFN2 and MFN1 situated on the OMM.^4^ Studies on models of Mitofusin 2 deletion have since then reported effects (often controversial) on MERC formation and/or stability, fueling debate about how MFN2 could simultaneously coordinate such diverse functions.^3^

**Fig. 1:**
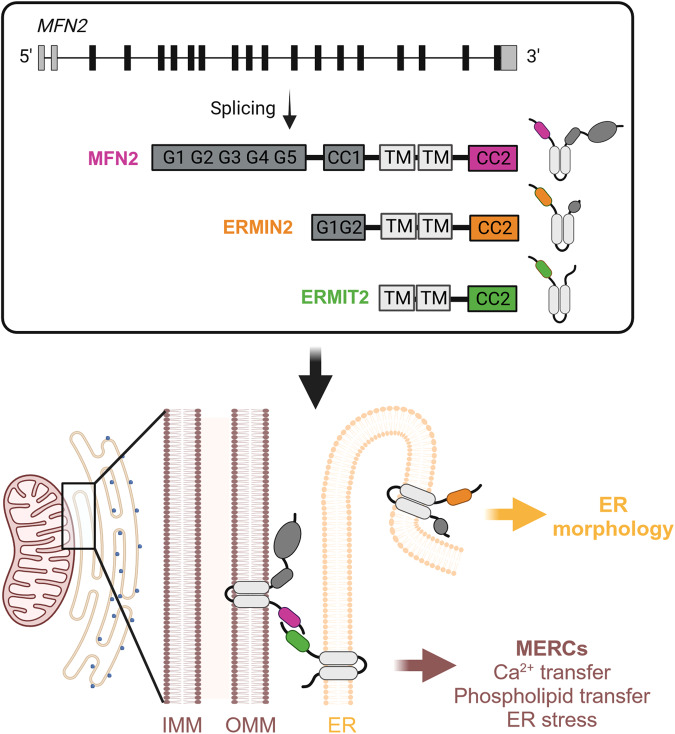
ER-specific functions of spliced MFN2 variants. Alternative splicing of *MFN2* produces ERMIN2 and ERMIT2 variants, who lack in full or in part the GTPase and coiled-coil 1 domains and localize to the ER. While ERMIT2 specifically stabilizes MERCs and associated functions via interaction with MFN2 or MFN1, ERMIN2 shapes ER architecture. Created with Biorender.com

Instead of pursuing the hypothesis of a promiscuous role of MFN2 itself, Naòn et al. explored the possibility that the *MFN2* gene may give rise to functionally distinct splice variants. They identified two such variants in the human allele that are significantly shorter than the corresponding full-length *MFN2* transcript, as they lack (fully or in part) the coiled-coil 1 and the GTPase domains (Fig. 1). Not only are these variants translated into proteins, but they exhibited a specific localization to the ER, hence they were termed *ERMIN2* and *ERMIT2*. Topologically, both variants exhibit their N and C termini exposed to the cytosol, and none of them could rescue the morphological defects of the mitochondrial network which characterizes *Mfn2* knockout cells. However, ERMIN2 appeared able to specifically rescue ER morphology, while ERMIT2 was necessary to restore ER-mitochondria tethering in the presence of MFN1. MERC functionality following ERMIT2 expression was validated in cells in vitro by monitoring mitochondrial Ca^2+^ uptake following ER release, and in liver-specific *Mfn2* knockout mice by tracing L-serine incorporation into phosphatidylserine and phosphatidylethanolamine (as a proxy to evaluate ER-to-mitochondria lipid transfer). Intriguingly, viral-mediated ERMIT2 expression alone in liver-specific *Mfn2* knockout mice or in a model of diet-induced NASH (nonalcoholic steatohepatitis) improved hepatic inflammation and reduced the expression of ER stress markers,^3^ supporting the specific role of MERCs in liver metabolism.

This study provides fundamental insights into the biological function of *MFN2* and associated splice variants, disentangling their specific roles in regulating mitochondrial and ER dynamics, but also tethering functions. It is of particular interest that in contrast to human *MFN2*, only one mouse *Mfn2* variant (MoV-*Mfn2*) was identified, which appears to be an analog of *ERMIT2*. This would argue that the ER-shaping function of ERMIN2 might be taken over by MoV-Mfn2 in mouse, suggesting that the emergence of an additional species-specific *MFN2* variant (*ERMIN2*) may play important roles in e.g., disease or higher-order functions in humans. On the other hand, this work also raises several questions. For instance, given the similar structure and topology of the two splice variants, it remains unclear why MERCs are stabilized uniquely by ERMIT2, despite the ability of Mitofusins to bind both, ERMIN2 and ERMIT2.^3^ Likewise, no mechanistic explanation was yet provided about how ERMIN2 may carry out its proposed function on ER remodeling. Is this function required for both, ER tubules and sheets, or is rather specific for certain domains? And how exactly does ERMIN2 exert this function? Is this role played via homotypic interactions or by interacting with other partners on the ER or on other organelles distinct from mitochondria? Given the pathophysiological role of *MFN2* missense mutations in causing Charcot-Marie-Tooth 2A (CMT2A), a severe form of peripheral axonopathy, it will be important for future work to assess the relevance of these newly discovered variants for neuronal and axonal integrity, particularly in settings of loss- or gain-of-function mutations affecting *ERMIN2* or *ERMIT2*-related functions that may cause previously neglected ER-specific phenotypes.^5^ In view of the often-conflicting evidence obtained from available genetic mouse models of CMT2A, which poorly reflect the clinical traits affecting CMT2A patients, the findings presented in this study thus pave the way for the generation of new pre-clinical models that may more faithfully reveal species-specific effects linked to dysregulated *MFN2* splice-variants during disease.
